# Supplementary material for: Salinity Alleviation and Reduction in Oxidative Stress by Endophytic and Rhizospheric Microbes in Two Rice Cultivars
Source: Plants (Basel). 2023 Feb 21;12(5):976. doi: 10.3390/plants12050976 (PMC10005660; doi:10.3390/plants12050976)
Supplement: Supplementary file 1 [file plants-12-00976-s001.zip › plants-2204968-supplementary.pdf]

Table S1. List of RT-PCR primers used in the study.

S. No.	Oligo (5'- 3')	Details	Reference
1.	TCCATCTTGGCATCTCTCAG	*Actin*-F	Wang et al., 2010 [43]
2.	GTACCCTCATCAGGCATCTG	*Actin*-R	Wang et al., 2010 [43]
3.	TACATGGGCAATGGCGGT	*OsPIP*1*;*1-*F*	Liu et al., 2006 [44]
4.	CAAGACCGTCACCCTTGGTG	*OsPIP*1*;*1-*R*	Liu et al., 2006 [44]
5.	AGCTCAAACAAGTCAAGAGC	*DHN* -F	Kumar et al., 2014 [16]
6.	AAGCACCAAACTAACACACG	*DHN* -R	Kumar et al., 2014 [16]
7.	GGAGGCCATGTCAATCATTC	*MnSOD*1-F	Kim et al., 2004 [45]
8.	CACAAGGTCCAGAAGTGCAA	*MnSOD*1-R	Kim et al., 2004 [45]
9.	GTTTTGAGGGACCTTGGACA	*cAPXa*-F	Kim et al., 2004 [45]
10.	TAACAGCCCACCGAGACATT	*cAPXa*-R	Kim et al., 2004 [45]
11.	CCACCACAACAACCACTACG	*CATa*-F	Kim et al., 2004 [45]
12.	CCAACGACTCATCACACTGG	*CATa*-R	Kim et al., 2004 [45]
13.	GAGTGAGGAGCTCATTGTTTACGA	*SERF*1-F	Formentin et al., 2018 [15]
14.	ACATCAAAATTTCCATGTCATCTA	*SERF*1-R	Formentin et al., 2018 [15]
